# Supplementary figures and images for: The m6A reader protein YTHDF2 facilitates HTLV-1 infectious and mitotic propagation by stabilizing Tax RNA
Source: J Virol. 2026 May 15;100(6):e00529-26. doi: 10.1128/jvi.00529-26 (PMC13288785; doi:10.1128/jvi.00529-26)

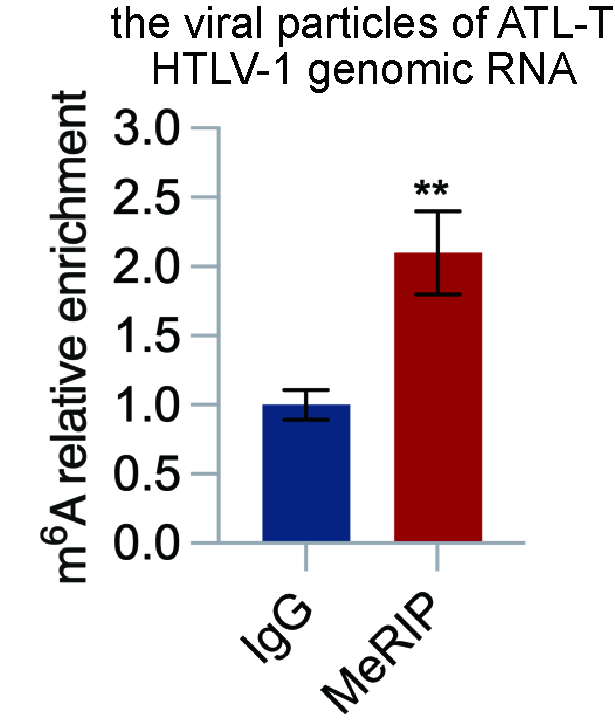

Supplement: Fig. S1 — HTLV-1 genomic RNA is m6A modified. [file jvi.00529-26-s0001.tif]

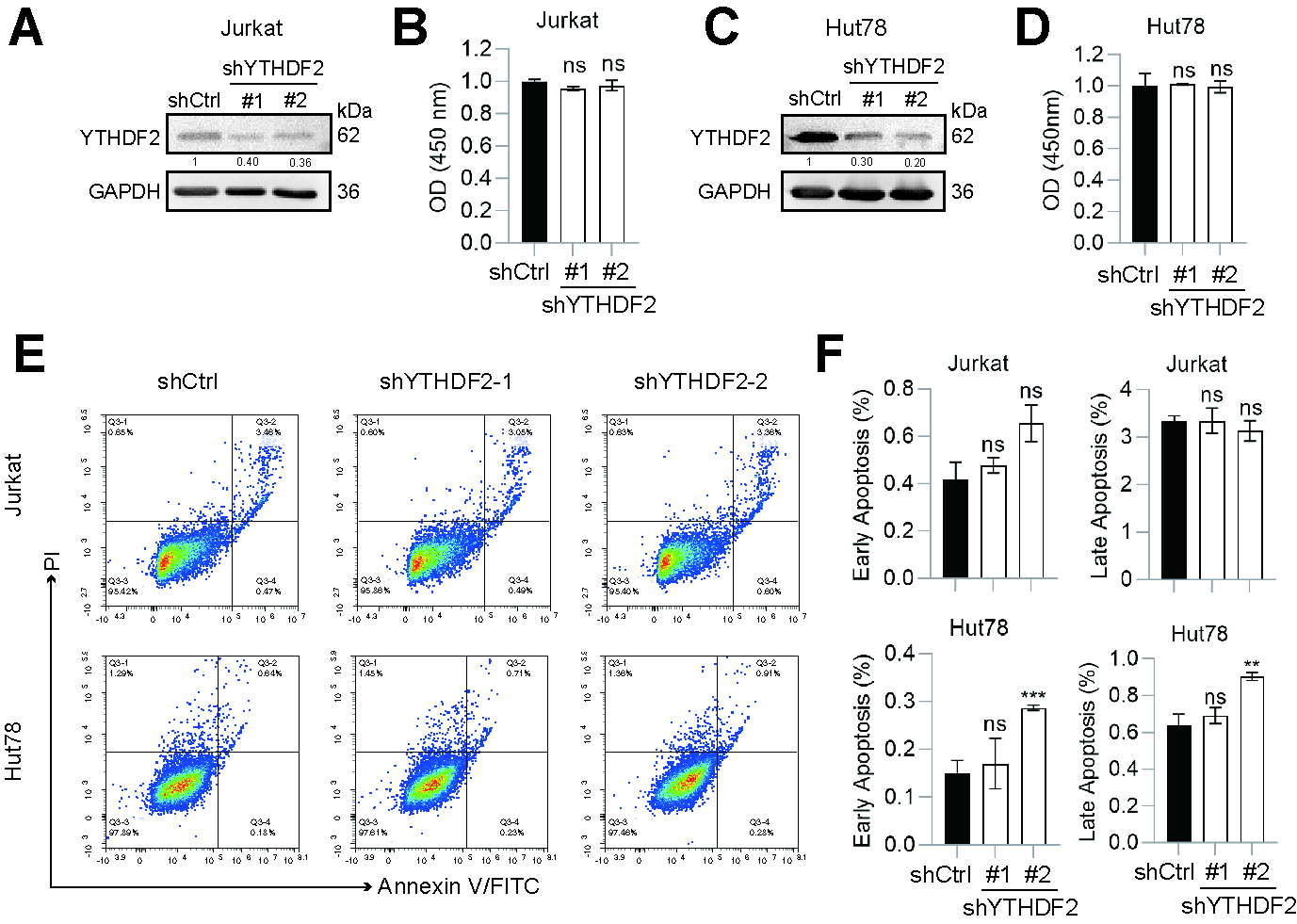

Supplement: Fig. S2 — Effects of YTHDF2 knockdown on proliferation and apoptosis in HTLV-1 negative Jurkat and Hut78 cells. [file jvi.00529-26-s0002.tif]

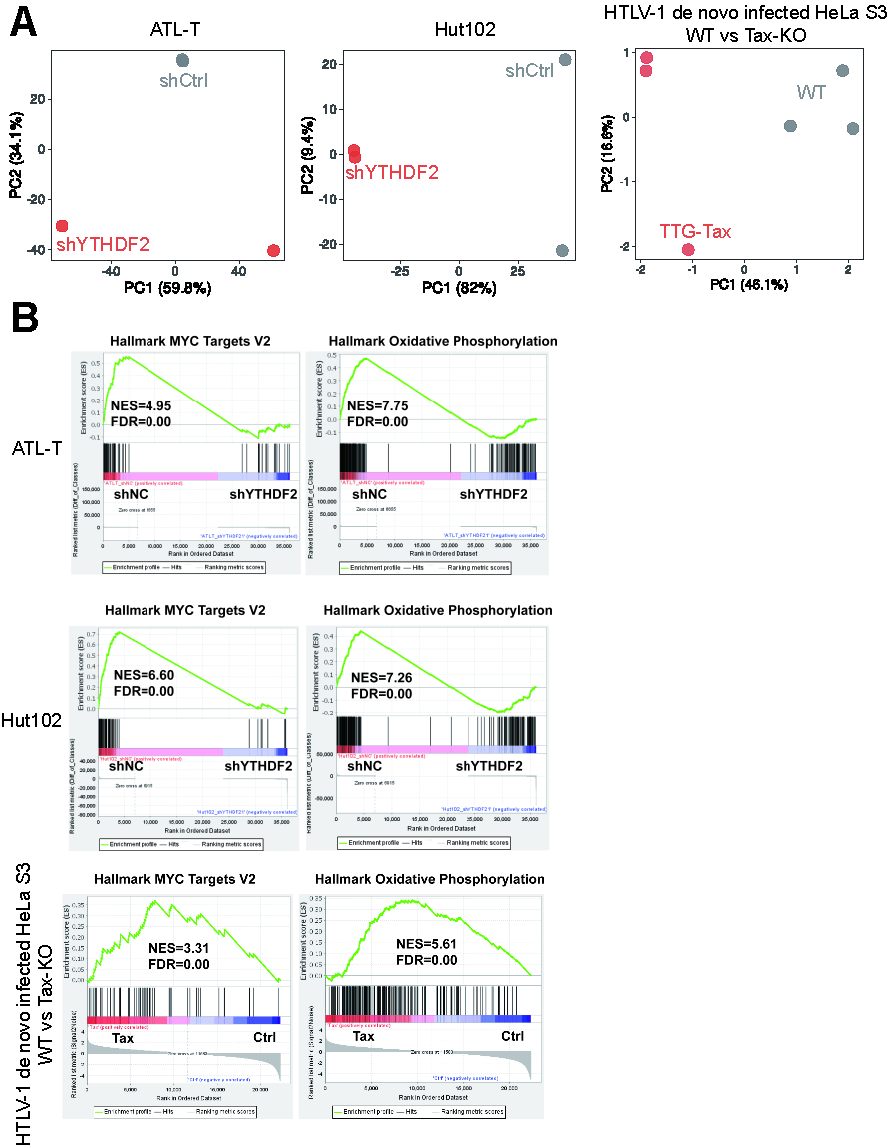

Supplement: Fig. S3 — Transcriptomic results show YTHDF2 and Tax regulate overlapping pathways. [file jvi.00529-26-s0003.tif]

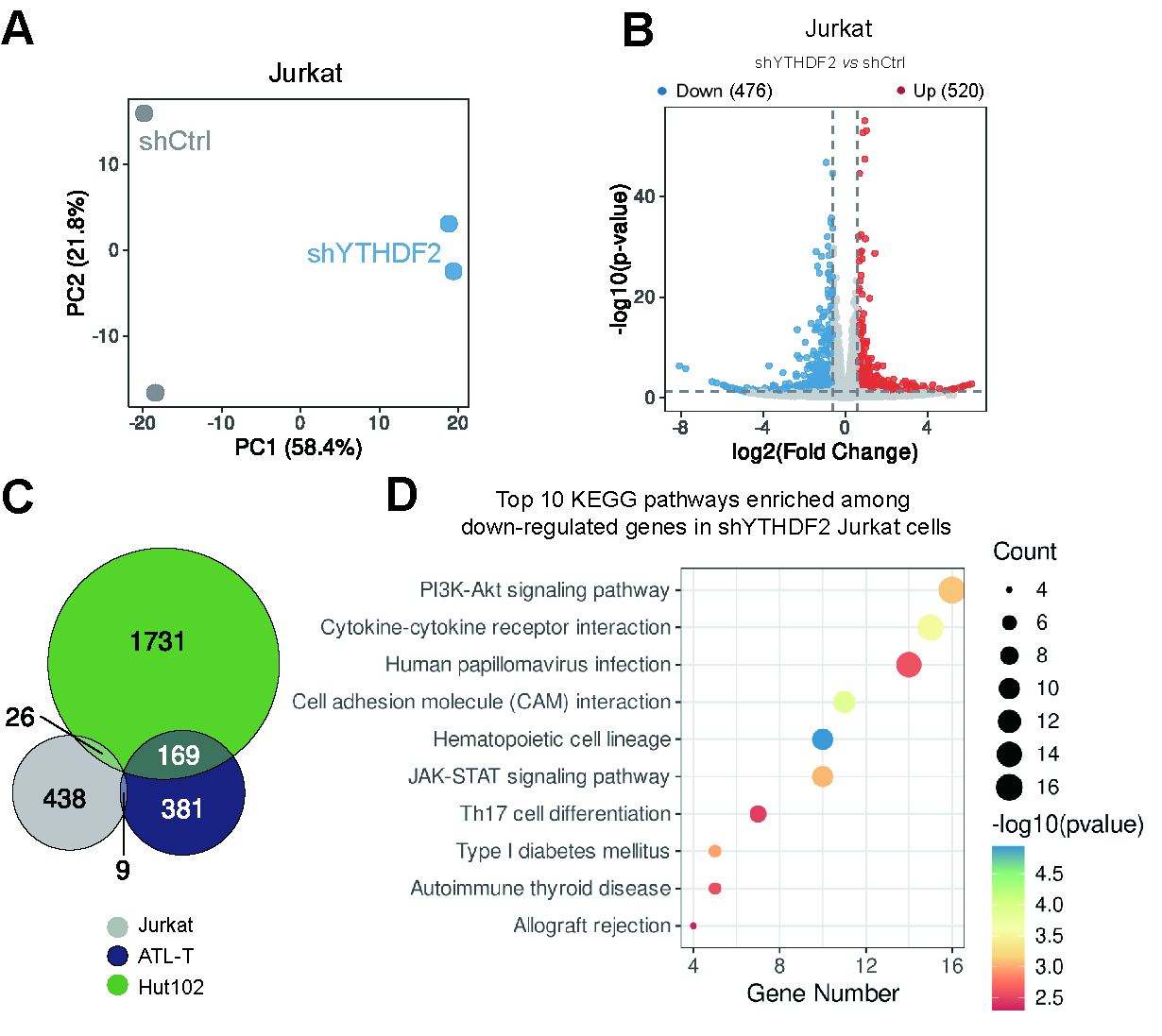

Supplement: Fig. S4 — Distinct regulatory roles of YTHDF2 in HTLV-1-infected and uninfected T cells. [file jvi.00529-26-s0004.tif]

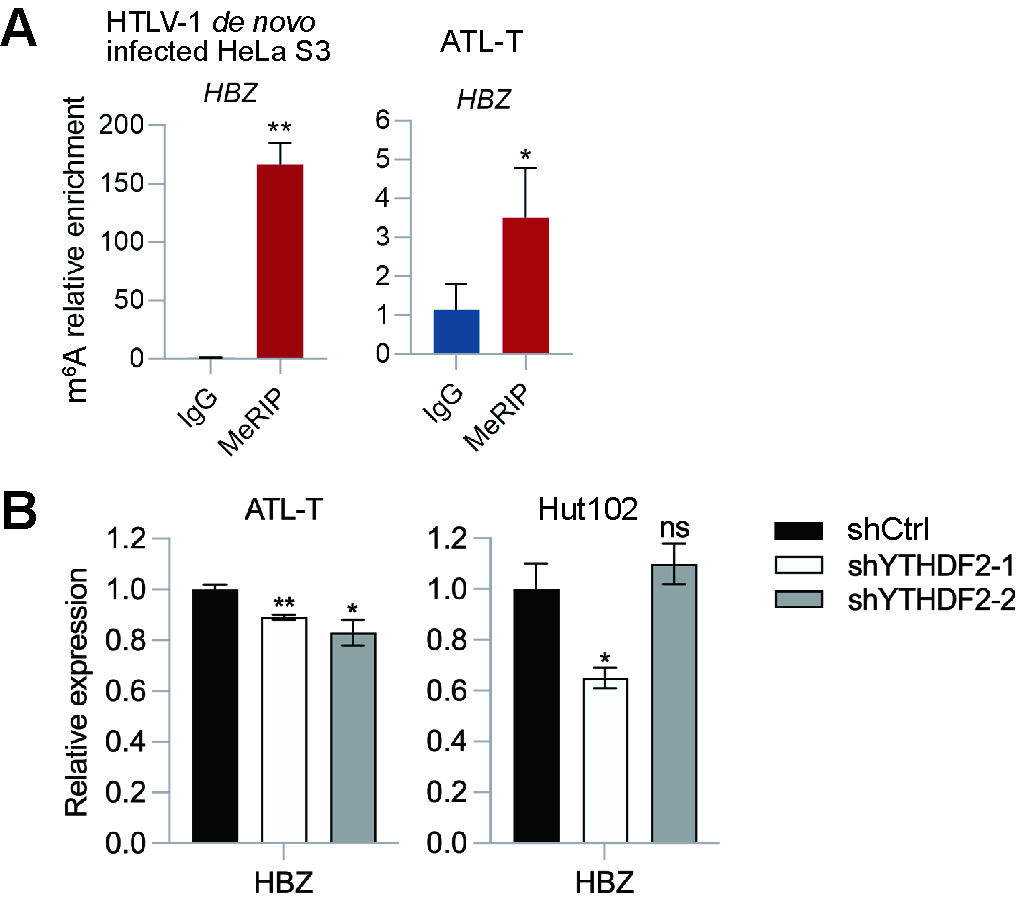

Supplement: Fig. S5 — HTLV-1 HBZ RNA is m6A modified. [file jvi.00529-26-s0005.tif]
