# Supplementary material for: The m6A reader protein YTHDF2 facilitates HTLV-1 infectious and mitotic propagation by stabilizing Tax RNA
Source: J Virol. 2026 May 15;100(6):e00529-26. doi: 10.1128/jvi.00529-26 (PMC13288785; doi:10.1128/jvi.00529-26)
Supplement: Supplemental legends — Descriptive legends for Fig. S1 to S5. [file jvi.00529-26-s0006.docx]

**Supplemental Figures**

**Fig. S1 HTLV-1 genomic RNA is m^6^A modified.**

MeRIP-qPCR analysis detected m^6^A modifications of HTLV-1 genomic RNA in viral particles of ATL-T. *P* value was calculated using a two-tailed unpaired Student's t-test. ^**^p<0.01. The results are representatives of three independent experiments.

**Fig. S2 Effects of YTHDF2 knockdown on proliferation and apoptosis in HTLV-1 negative Jurkat and Hut78 cells.**

(A and C) Immunoblot analysis of YTHDF2 knockdown efficiency in (A) Jurkat and (C) Hut78 cells, with GAPDH used for normalization. (B and D) Cell proliferation was measured by CCK-8 assay at 72 h after YTHDF2 knockdown in (B) Jurkat and (D) Hut78 cells. (E) Flow cytometry analysis of apoptosis in (*Upper*) Jurkat and (*Lower*) Hut78 cells after YTHDF2 knockdown. (F) Statistical analysis of early and late apoptosis based on the data in (E). *P* value was calculated using a two-tailed unpaired Student's t-test. ^**^p<0.01, ^***^p<0.00. ns, not significant. The results are representatives of three independent experiments.

**Fig. S3 Transcriptomic results show YTHDF2 and Tax regulate overlapping pathways.**

(A) PCA of RNA-seq from YTHDF2 knockdown ATL-T, Hut102 and HTLV-1 de novo infected HeLa S3 cells. (B) GSEA demonstrating that hallmark genes associated with MYC targets V2 and Oxidative phosphorylation genes sets are promoted by endogenous YTHDF2 in ATL-T and Hut102 and Tax in HTLV-1 de novo infected HeLa S3 cells. FDR < 0.05* for all signatures.

**Fig. S4 Distinct regulatory roles of YTHDF2 in HTLV-1-infected and uninfected T cells.**

(A) PCA of RNA-seq from YTHDF2 knockdown Jurkat cells. (B) The volcano plot illustrates differentially expressed genes regulated by YTHDF2 in Jurkat cells. (C) Venn diagram showing the overlap of downregulated genes in Hut102, ATL-T, and Jurkat cells upon YTHDF2 knockdown. (D) KEGG pathway enrichment of downregulated genes upon YTHDF2 knockdown in Jurkat cells.

**Fig. S5 HTLV-1 HBZ RNA is m^6^A modified.**

(A) MeRIP-qPCR analysis detected m^6^A modifications of HBZ RNA in (*Left*) HTLV-1 infected HeLa S3 cells and (*Right*) ATL-T cells. (B) Following YTHDF2 knockdown via shRNA in in (*Left*) ATL-T and (*Right*) Hut102 cells, qPCR was used to quantify the expression levels of HBZ. *P* value was calculated using a two-tailed unpaired Student's t-test. ns, not significant; ^*^p<0.05, ^**^p<0.01. The results are representatives of three independent experiments.

**Supplemental Tables**

**Table S1. Prediction of m^6^A Modification Sites in HTLV-1 RNA (refJ02029) Using SRAMP.**

**Table S2. Primers of MeRIP-qPCR for HTLV-1 RNAs.**

**Table S3. Primers of RT-qPCR.**
